# Draft Genome Sequence of Toxigenic *Corynebacterium ulcerans* Strain 03-8664 Isolated from a Human Throat

**DOI:** 10.1128/genomeA.00719-16

**Published:** 2016-07-28

**Authors:** Luis C. Guimarães, Marcus V. C. Viana, Leandro J. Benevides, Diego C. B. Mariano, Adonney A. O. Veras, Pablo H. C. Sá, Flávia S. Rocha, Priscilla C. B. Vilas Boas, Siomar C. Soares, Maria S. Barbosa, Nicole Guiso, Edgar Badell, Vasco Azevedo, Rommel T. J. Ramos, Artur Silva

**Affiliations:** a Institute of Biological Sciences, Federal University of Pará (UFPA), Belém, Pará, Brazil; b Institute of Biological Sciences, Federal University of Minas Gerais (UFMG), Belo Horizonte, Minas Gerais, Brazil; c Department of Immunology, Microbiology and Parasitology, Institute of Biological Sciences and Natural Sciences, Federal University of Triângulo Mineiro (UFTM), Uberaba, Minas Gerais, Brazil; d Institut Pasteur, Unité de Prévention et Thérapies Moléculaires des Maladies Humaines, National Centre of Reference of Toxigenic Corynebacteria, Paris, France

## Abstract

*Corynebacterium ulcerans* is an emergent pathogen infecting wild and domesticated animals worldwide that may serve as reservoirs for zoonotic infections. In this study, we present the draft genome of *C. ulcerans* strain 03-8664. The draft genome has 2,428,683 bp, 2,262 coding sequences, and 12 rRNA genes.

## GENOME ANNOUNCEMENT

The *Corynebacterium ulcerans tox* gene was first described in 1926, isolated from a human throat (1). The *tox* gene present in *C. ulcerans* has 95% similarity compared to the *tox* gene present in *Corynebacterium diphtheriae* (2). This gene encodes diphtheria toxin present in lysogenic β-corynephages described in *C. ulcerans* 0102 but not in *C. ulcerans* 809 (both strains available at GenBank) (3).

However, the virulence of *C. ulcerans* does not necessarily depend on the production of diphtheria toxin; some strains have been reported to produce a powerful and severe dermonecrotic toxin similar to phospholipase D from *Corynebacterium pseudotuberculosis* (4). This repertoire of potent toxins shared by *C. ulcerans*, *C. diphtheriae*, and *C. pseudotuberculosis*, along with levels of genomic DNA relatedness and taxonomic analyzes of 16S rRNA gene sequences, particularly highlights the close phylogenetic relationship, putting these three species in a distinct cluster of the genus *Corynebacterium* (5).

*C. ulcerans* is an emergent pathogen infecting wild and domesticated animals that may serve as reservoirs for zoonotic infections. The frequency and severity of human infections reported worldwide during the past two decades have increased its medical importance (5, 6). Nevertheless, little knowledge about the lifestyle and associated virulence factors of *C. ulcerans* was available until recently. In humans, it may cause diphtheria-like disease, pharyngitis, sinusitis, tonsillitis, pulmonary nodules, and skin ulcers (7).

In this study, we present the draft genome sequence of toxigenic *Corynebacterium ulcerans* strain 03-8664. This strain was isolated from a human throat in France. The strain is part of the Collection of Institut Pasteur (CIP) (https://www.pasteur.fr/en) and was kindly given to the Laboratory of Genomics and System Biology located at Federal University of Pará, Belém, Pará, Brazil, and the Laboratory of Cellular and Molecular Genetics Federal University of Minas Gerais, Belo Horizonte, Minas Gerais, Brazil.

The SOLiD platform was used to perform the genome sequencing, using a fragment library. The predicted genome coverage was approximately 1,900× based on *C. ulcerans* genomes available in GenBank (http://www.ncbi.nlm.nih.gov/genbank/). The *de novo* assembly strategy was performed through SOAP*denovo* version 2.04 (8) and Velvet version 1.0.13 (9). The assembly generated 258 contigs with 2,428,683 bp. The contigs were submitted to GenBank for automatic annotation. The genome has 2,270 coding sequences, 12 rRNA genes, 49 tRNA genes, 272 pseudogenes, and a G+C content of 53.60%. This genome is part of further studies of comparative genomics, pathogenicity, and vaccine and drug targets of the species.

### Nucleotide sequence accession numbers.

The *C. ulcerans* whole-genome shotgun (WGS) project has the project accession number LGSY00000000. This version of the project has the accession number LGSY02000000, and consists of sequences LGSY02000001 to LGSY02000258.

## References

[1] Gulbert R, Stewart FC 1926 *Corynebacterium ulcerans*: a pathogenic microorganism resembling *C. diphtheriae*. J Lab Clin Med 12:756–761.

[2] Sing A, Bierschenk S, Heesemann J 2004 Classical diphtheria caused by *Corynebacterium ulcerans* in Germany: amino acid sequence differences between diphtheria toxins from *Corynebacterium diphtheriae* and *C. ulcerans*. Clin Infect Dis 40:325–326. 15655760 10.1086/426687

[3] Sekizuka T, Yamamoto A, Komiya T, Kenri T, Takeuchi F, Shibayama K, Takahashi M, Kuroda M, Iwaki M 2012 *Corynebacterium ulcerans* 0102 carries the gene encoding diphtheria toxin on a prophage different from the *C. diphtheriae* NCTC 13129 prophage. BMC Microbiol 12:72. doi:10.1186/1471-2180-12-72. 22583953 PMC3406963

[4] McNamara PJ, Cuevas WA, Songer JG 1995 Toxic phospholipases D of *Corynebacterium pseudotuberculosis*, *C. ulcerans* and *Arcanobacterium haemolyticum*: cloning and sequence homology. Gene 156:113–118. doi:10.1016/0378-1119(95)00002-N. 7737503

[5] Trost E, Al-Dilaimi A, Papavasiliou P, Schneider J, Viehoever P, Burkovski A, Soares SC, Almeida SS, Dorella FA, Miyoshi A, Azevedo V, Schneider MP, Silva A, Santos CS, Santos LS, Sabbadini P, Dias AA, Hirata R, Mattos-Guaraldi AL, Tauch A 2011 Comparative analysis of two complete *Corynebacterium ulcerans* genomes and detection of candidate virulence factors. BMC Genomics 12:383. 21801446 10.1186/1471-2164-12-383 PMC3164646

[6] Dias AA, Santos LS, Sabbadini PS, Santos CS, Silva Júnior FC, Napoleão F, Nagao PE, Villas-Bôas MH, Hirata Júnior R, Guaraldi AL 2011 *Corynebacterium ulcerans* diphtheriae: an emerging zoonosis in Brazil and worldwide. Rev Saúde Publica 45:1176–1191. 22124745 10.1590/s0034-89102011000600021

[7] Bernard K 2012 The genus *Corynebacterium* and other medically relevant coryneform-like bacteria. J Clin Microbiol 50:3152–3158. doi:10.1128/JCM.00796-12. 22837327 PMC3457441

[8] Li R, Zhu H, Ruan J, Qian W, Fang X, Shi Z, Li Y, Li S, Shan G, Kristiansen K, Li S, Yang H, Wang J, Wang J 2010 *De novo* assembly of human genomes with massively parallel short read sequencing Genome Res 20:265–272. 20019144 10.1101/gr.097261.109 PMC2813482

[9] Zerbino DR, Birney E 2008 Velvet: algorithms for *de novo* short read assembly using de Bruijn graphs. Genome Res 18:821–829. doi:10.1101/gr.074492.107. 18349386 PMC2336801

